# Mitochondrial genome of the *Melophus lathami*

**DOI:** 10.1080/23802359.2016.1250129

**Published:** 2016-12-09

**Authors:** Yu Zhang, Kai Li, Tao Pan, Zhaojie Peng, Xiaonan Sun, Xing Kang, Yanan Zhang, Baowei Zhang

**Affiliations:** School of Life Sciences, Anhui University, Hefei, Anhui, PR China

**Keywords:** *Melophus lathami*, mitochondrial genome, phyolgenetic tree

## Abstract

Crested Bunting (*Melophus lathami*) is the single species of the *Melophus*, which was mainly distributed to the south of Asia. The complete sequence of the mitochondrial DNA of *M. lathami* (16,799bp in length) have been obtained, which consists of 13 protein-coding genes, 22 tRNA genes, two rRNA genes (12S rRNA and 16S rRNA), and one control region (D-loop). The nucleotide composition was 30.1% A, 33.0% C, 14.3% G, and 22.6% T. Besides, the phylogenetic tree based on 12S and 16S rRNA was divided into three clades. *M. lathami* was determined in the second lineage.

The Crested Bunting (*Melophus lathami*) is a passerine bird with widespread in southern Asia (MacKinnon & Phillipps 2000). Compared to the moist environment, *M. lathami* prefer open and dry areas. They usually perch on farmland and foothills of rocky slopes, are also found in urban and rural areas. According to The IUCN Red List of Threatened Species, it was listed as least concern (IUCN2016). *M. lathami* is a large dark bunting (17 cm in length) with elongated head crest (MacKinnon & Phillipps 2000). For *M. lathami*, which is so divergent from all other *Emberiza* lies in both in plumage and in having a prominent crest on the crown (Alstrom et al. 2008).

The *M. lathami* sample was collected in shanghai, China in March, 2016. Now, the specimen was stored in the Key Laboratory of Eco-engineering and Bio-technique, School of Life Sciences, Anhui University. In our study, the complete mtDNA of *M. lathami* was amplified using 18 pairs of primers by polymerase chain reaction (PCR). We sequenced data by MEGA 6.0 (Tamura et al. 2013) and DnaSP v5 (Librado & Rozas 2009), and submitted the complete sequence of the mitochondrial DNA (mtDNA) to GenBank (accession number KX702277).

Circular complete mtDNA sequence of *M. lathami* is 16,799bp in length. The overall base composition of this mtDNA was 30.1% A, 33.0% C, 14.3% G, and 22.6% T. The complete mtDNA sequence of *M. lathami* had 13 protein-coding genes, 22 tRNA genes, two rRNA genes (12S rRNA and 16S rRNA), and one control region. All the protein-coding genes in *M. lathami* are distributed on the H-strand, except the ND6 subunit gene, rep-origin, and eight tRNA genes (*tRNA^Gln^*, *tRNA^Ala^*, *tRNA^Asn^*, *tRNA^Cys^*, *tRNA^Tyr^*, *tRNA^Ser^*^(UCN)^, *tRNA^Pro^*, *tRNA^Glu^*), which are encoded on the L-strand. In 13 protein-coding genes, the shortest one is ATP8 gene (168 bp) and the longest one is the ND5 gene (1818 bp). For most protein-coding genes, they take ATG as start codons, while ND3 regard ATA as start codons. The control region (D-loop) of the *M. lathami* mtDNA was 1212 bp in length.

We constructed the phylogenetic tree by Bayesian inference (BI) with MrBayes 3.2 (Ronquist et al. 2012). In our study, the tree based on 12S and 16S rRNA included nine kinds of *Emberiza*, *M. lathami* and *Passer montanus*. *Passer montanus* was used as outgroup. As shown in Figure 1, the phylogenetic tree was divided into three major clades. *E. elegans* makes up the first lineage. The second lineage includes three species (*Emberiza jankowskii*; *E. cioides*; *Melophus lathami*). The third lineage is composed by *E. rutila*, *E. aureola*, *E. rustica*, *E. pusilla*, *E. tristrami,* and *E. chrysophrys*. *M. lathami* lies in a separate small branch, in general, it presented a relative close relationship with *Emberiza* at the molecular level, especially *E. jankowskii* and *E. cioides*.

**Figure 1.**
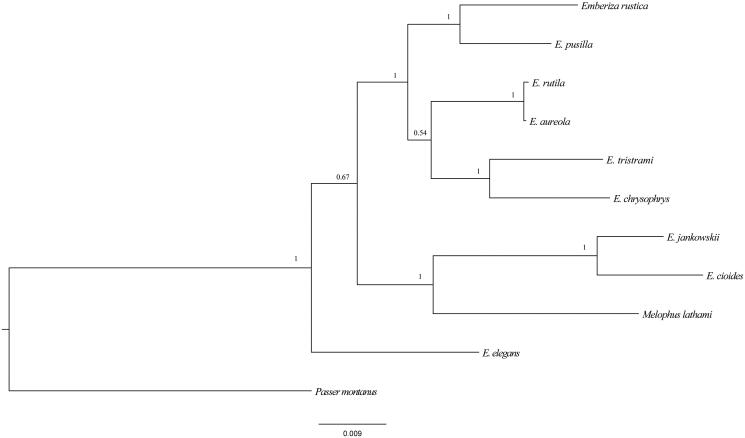
Phylogenetic tree based on 12S and 16S sequences using Bayesian inference (BI) with MyBays 3.2 GenBank accession number for the published sequences: *E. pusilla*: KC 407232; *E. tristrami*: NC 015234.1; *E. chrysophrys*: NC 015233; *E. rustica*: KC 831775; *E. chrysophrys*: HQ896034; *E. rutila*: KC 952874; *E. aureola*: KF 111713; *E. cioides*: KF 322027; *E. Jankowskii*: KP738714; *Melophus lathami*: KX702277; *Passer montanus*: NC024821.

Mitochondrial DNA, as a powerful and important way to explore genome evolution, helps to infer ancient evolutionary relationships (Boore 1999; Pan et al. 2015; Sun et al. 2016). We expect our study to provide useful database for further study in population evolution and phylogenetic relationship.
